# Sex differences and similarities in the neuroimmune response to central administration of poly I:C

**DOI:** 10.1186/s12974-021-02235-7

**Published:** 2021-09-06

**Authors:** Caitlin K. Posillico, Rosa E. Garcia-Hernandez, Natalie C. Tronson

**Affiliations:** Psychology Department, University of Michigan, 530 Church St., Ann Arbor, MI 48109 USA

**Keywords:** Poly I:C, Sex differences, Neuroimmune, Cytokines, Hippocampus, Chemokines, Male, Female, Interferon, Mouse

## Abstract

**Background:**

The neuroimmune system is required for normal neural processes, including modulation of cognition, emotion, and adaptive behaviors. Aberrant neuroimmune activation is associated with dysregulation of memory and emotion, though the precise mechanisms at play are complex and highly context dependent. Sex differences in neuroimmune activation and function further complicate our understanding of its roles in cognitive and affective regulation.

**Methods:**

Here, we characterized the physiological sickness and inflammatory response of the hippocampus following intracerebroventricular (ICV) administration of a synthetic viral mimic, polyinosinic:polycytidylic acid (poly I:C), in both male and female C57Bl/6N mice.

**Results:**

We observed that poly I:C induced weight loss, fever, and elevations of cytokine and chemokines in the hippocampus of both sexes. Specifically, we found transient increases in gene expression and protein levels of IL-1α, IL-1β, IL-4, IL-6, TNFα, CCL2, and CXCL10, where males showed a greater magnitude of response compared with females. Only males showed increased IFNα and IFNγ in response to poly I:C, whereas both males and females exhibited elevations of IFNβ, demonstrating a specific sex difference in the anti-viral response in the hippocampus.

**Conclusion:**

Our data suggest that type I interferons are one potential node mediating sex-specific cytokine responses and neuroimmune effects on cognition. Together, these findings highlight the importance of using both males and females and analyzing a broad set of inflammatory markers in order to identify the precise, sex-specific roles for neuroimmune dysregulation in neurological diseases and disorders.

## Background

The neuroimmune system is responsible for surveying the microenvironment and responding to illness, injury, and infection. It is also required for behavioral responses to infection [1, 2] and normal, non-immune neural processes [3–5] including synaptic plasticity and memory formation [6, 7]. Neuroimmune activity in the hypothalamus has a well-described role in physiological responses (e.g., febrile response), and activation in the hippocampus also triggers sickness behaviors, depression-like symptoms, acute impairments of learning and memory [3, 8], and long-lasting changes in cognitive function [9, 10].

Activation of immune signaling in the hippocampus has been implicated in disorders of affect and cognition, many of which show sex-biases in prevalence and outcomes [8, 11, 12]. There is overwhelming evidence for sex differences in immune responses in the periphery [13, 14], but limited data on whether and how neuroimmune activation differs between males and females in adult brains. Understanding sex differences in the hippocampus is important for understanding exactly how neuroimmune activation impacts cognition and contributes to psychiatric and neurological disorders in both sexes.

Illness, injury, or aseptic triggers of the innate immune system—either bacterial endotoxins (e.g., lipopolysaccharide, LPS) or viral mimics (e.g., polyinosinic:polycytidylic acid, poly I:C)—cause activation of neuroimmune cells, including microglia and astrocytes, and rapid production of cytokines in the brain [15, 16]. Due to key roles in peripheral inflammation, the cytokines IL-1β [17–19], IL-6 [20–22], and TNFα [23–25] have been the focus of much of the research of neuroimmune function [26]. More recently, other cytokines, including interferons [11, 27], CCL2 [28, 29], and CXCL10 [27, 30, 31] also play critical roles in modulation of behavior, cognition, and affective states, suggesting that many cytokines play important roles in these processes.

Sex differences in immune and neuroimmune activity have also been reported. Females have a greater peripheral immune response compared with males [13]. In contrast, neuroimmune cells in vitro, including astrocytes derived from male cortical tissue, have a significantly greater reaction to inflammatory insults compared with female-derived cells [32, 33]. We have identified sex differences in the magnitude, time course, and pattern of cytokines activated in the hippocampus following peripheral LPS [34], and in the long-lasting impact of LPS on hippocampal function [9]. Thus, sex differences in neuroimmune responses specifically may be a contributing factor to sex differences in neural and cognitive processes and disorders.

Despite incredible advances in psychoneuroimmunology over the past decade, there are critical gaps in our knowledge that preclude a holistic understanding of neuroimmune function and its impacts on cognitive function. First, with some notable exceptions [28, 35, 36], studies have typically focused on a few inflammatory cytokines (e.g., IL-1β, IL-6, and TNFα) critical for neuroimmune activation and its effects on cognition. Yet, it is now clear that the massive, coordinated cytokine response observed in the periphery also occurs in the central nervous system [34, 37]. The roles played by other cytokines, and the sex-specific patterns of activation, are yet to be defined [26]. Second, the bulk of studies aimed at understanding neuroimmune activation and its behavioral sequelae have used the gram-negative bacterial shell and toll-like receptor 4 (TLR4) agonist LPS. Nevertheless, viral illnesses—including COVID-19—also trigger changes in behavior, cognition, and emotional states, and significant sex differences have been observed in the context of viral infections [13, 14, 38]—an issue that has been propelled to the forefront during the current COVID-19 pandemic [39, 40]. Given that viruses act through distinct toll-like receptors, their impact is likely mediated by a different, albeit overlapping, pattern of cytokine activation compared with LPS or bacterial triggers. Third, due to its relevance for disease states, many in vivo studies of neuroimmune function use a peripheral immune challenge. Here, neuroimmune activation is primarily driven by peripheral immune signals that infiltrate the brain [41]. This complicates the interpretation of whether sex differences in cytokine levels observed in the brain are due to indirect effects based on sex differences in peripheral immune response or to direct effect of sex differences in neuroimmune function.

In this study, we aimed to identify a broader set of inflammatory cytokines induced in the hippocampus by direct neuroimmune stimulation *via* central administration of poly I:C in both males and females. We focused on the hippocampus because elevation of hippocampal cytokines is associated with both disruption of memory processes [3, 17, 19, 42–45] and increased depression-like behaviors [46, 47]. Within the hippocampus, we focused on cytokines and chemokines that have previously been implicated in cognitive and affective dysfunction, including the commonly studied IL-1β, IL-6, IL-10, and TNF⍺ [48, 49]; as well as IL-4 [50, 51], IL-2 [46, 52], CXCL10 [31, 53], and CCL2 [53]; as well as virus-specific responses (IFNα and IFNβ [54];); and measures of generic microglial and astrocyte activation (CD11b and GFAP [55, 56];).

We demonstrate that poly I:C induces fever, weight loss, and changes in mRNA expression and protein levels of cytokines, chemokines, and markers of glial activation across a 24-h period in both sexes. Notably, only IFNα and IFNɣ showed male-specific patterns of activation after central poly I:C administration, and many cytokines and chemokines showed a greater magnitude increase in males compared with females. Whether these sex differences in neuroimmune activation contribute to sex differences in modulation of cognition and affect and subsequent prevalence of memory- and mood-related diseases and disorders is an important area of research for our ongoing studies.

## Methods

### Animals

Ninety-nine male and female 8–9-week-old C57B/6N mice from Envigo (Indianapolis, IN) were used in these experiments. For all experiments, mice were individually housed in standard mouse cages with ad libitum access to food and water in a room with maintained temperature and pressure under a 12:12-h light/dark cycle. All mice had at least 1 week of acclimation to the colony room prior to any manipulations. All protocols were approved by the Institutional Animal Care and Use Committee (IACUC).

### Stereotaxic surgeries

Bilateral guide cannulae (PlasticsOne, Roanoke, VA) targeting the lateral ventricles were implanted using standard stereotaxic methods (KOPF, Tujunga, CA) at the following coordinates relative to Bregma: ML ± 1.00 mm, AP 0.30 mm, DV − 2.50 mm. Animals were administered a pre-surgical analgesic (5 mg/kg Carprofen, subcutaneous) and anesthetized for surgery using an intraperitoneal injection of 250 mg/kg of Avertin (2,2,2-tribromoethanol) which maintained a surgical plane of anesthesia for the duration of the craniotomy. Bilateral holes were drilled into the skull at the above coordinates, and guide cannulae were implanted using dental cement. Animals were given a second dose of Carprofen (5 mg/kg, subcutaneous) 24 h after surgery to maintain a total of 48 h of analgesia. Mice were monitored daily for 10 days post-operative and were given at least 2 weeks to recover from surgery prior to use in experiments.

### Poly I:C administration

Polyinosinic:polycytidylic acid (poly I:C; Cat. No. P1530; Sigma-Aldrich, St. Louis, MO) was prepared according to the manufacturer’s instructions and sterile-filtered using a 0.22-μm filter prior to administration. For intracerebroventricular (ICV) administration, we infused 20 μg of poly I:C (2 μL of 10 μg/μL poly I:C) [57] or an equal volume of 0.9% sterile saline *via* the implanted guide cannula under brief isoflurane anesthesia.

### Sickness behavior assessment

To confirm the efficacy of the ICV dose of poly I:C, and the specific poly I:C used here [58], poly I:C-induced physiological measures of sickness in males and females were assessed. Body weights and rectal temperatures (RET-3; Physitemp, Clifton, NJ) were measured at 2, 4, 6, 12, 24, and 48 h following ICV administration of poly I:C (*n* = 10 male; *n* = 9 female) or sterile saline (*n* = 10 male; *n* = 8 female; Fig. 2A). Visual and behavioral measures of sickness (piloerections, squinted eyes, hunched posture, and low responsivity) were assessed throughout [59]. No changes in overt sickness behaviors were observed for any experiment (data not shown).

#### Statistical analysis of sickness behaviors

Analysis of body weight and temperature changes in response to poly I:C was completed using a mixed repeated-measures ANOVA, using time post-infusion as the within-subjects factor and treatment and sex as the between-subjects factors with Greenhouse-Geisser corrections for sphericity. Significant main effects and interactions (*p* < 0.05) were followed up using post hoc tests with Bonferroni corrections for multiple comparisons, and effect sizes were calculated using the partial eta squared method. Any outliers were identified as samples outside the range of 2 standard deviations from the group mean.

### Characterization of the acute hippocampal neuroimmune response

We used RNA and protein endpoints to examine induction of cytokines and glial activation markers in the hippocampus. Males and females were treated with either poly I:C (*n* = 22 male; *n* = 24 female) or sterile saline (*n* = 8/sex) and brains were collected 0.5 h (*n* = 5 male; *n* = 6 female), 2 h (*n* = 6/sex), 4 h (*n* = 5 male; *n* = 6 female), and 24 h (*n* = 6/sex) later. All animals were transcardially perfused with 0.1 M phosphate buffer to remove circulating blood from the brain. Both hemispheres of dorsal hippocampus tissue were collected in separate RNase-/DNase-free, sterile microcentrifuge tubes, and immediately flash frozen. All samples were stored at – 80°C before tissue processing.

#### Quantitative real-time PCR

One hemisphere of dorsal hippocampal tissue per mouse was processed for gene expression analysis using quantitative real-time PCR. Frozen samples were homogenized, and messenger RNA (mRNA) was extracted (PureLink RNA Mini Kit; Cat. No. 12183020; Invitrogen, Carlsbad, CA) under sterile, RNase-free conditions. RNA quality was assessed using gel electrophoresis, and UV spectroscopy was used to assess RNA purity (A260/280 > 1.80) and quantity (BioSpectrometer Basic; Eppendorf, Hamburg, Germany). Any genomic DNA in the sample was removed using DNase treatment, and 800 ng of cDNA was synthesized from each mRNA sample (QuantiTect Reverse Transcriptase Kit; Cat. No. 205314; Qiagen, Hilden, Germany). Any samples that did not have a high enough concentration of RNA to make 800 ng of cDNA were removed from further analyses (*n* = 3 male; *n* = 5 female). Relative gene expression was measured using Power SYBR Green PCR Master Mix (Cat. No. 4368702; Applied Biosystems, Foster City, CA) in 10 μL reactions (ABI 7500 real-time PCR system; Cat. No. 4351105; Applied Biosystems).

We measured expression of four commonly used housekeeping genes: *18s*, *gapdh*, *hprt1*, and *rplp0* (all QuantiTect Primer Assays: *18s* Cat. No. QT02448082, *gapdh* Cat. No. QT01658692, *hprt1* Cat. No. QT00166768, *rplp0* Cat. No. QT00249375; Qiagen). We analyzed the relative expression of the following genes of interest: *ccl2*, *cd11b*, *cxcl10*, *gfap*, *ifnα*, *ifnβ*, *ifnγ*, *il-1α*, *il-1β*, *il-6*, *il-10*, and *tnfα*. The gene primer for *il-1α* was a QuantiTect Primer Assay (Cat. No. QT00113505; Qiagen). The sequences for the remaining gene primers can be found in Table 1 and were ordered through Integrated DNA Technologies and diluted to 0.13 μM to be used for PCR. All Qiagen primers were diluted as per the manufacturer’s instruction.

**Table 1:** Primer sequences used for real-time PCR

Gene target	Forward primer sequence (5′ to 3′)	Reverse primer sequence (5′ to 3′)	NCBI reference sequence
***ccl2***	CCACAACCACCTCAAGCACT	AAGGCATCACAGTCCGAGT	NM_011333.3
***cd11b***	CGTGAATGGGGACAAACTGAC	GCACTGAGGCTGGCTATTGA	NM_008401.2
***cxcl10***	TCCATCACTCCCCTTTACCCA	TGGCTTGACCATCATCCTGC	NM_021274.2
***gfap***	AAACCGCATCACCATTCCTG	CCCGCATCTCCACAGTCTTTA	NM_010277.3
***ifnα***	AGAGAAGAAACACAGCCCCT	AGCACATTGGCAGAGGAAGA	NM_010502.2
***ifnβ***	GCTCCAAGAAAGGACGAACAT	GGATGGCAAAGGCAGTGTAA	NM_010510.1
***ifnγ***	GTCAACAACCCACAGGTCCA	CGACTCCTTTTCCGCTTCCT	NM_008337.4
***il-1β***	TGCCACCTTTTGACAGTGATG	GCTCTTGTTGATGTGCTGCT	NM_008361.4
***il-6***	GAGACTTCCATCCAGTTGCCT	TCATTTCCACGATTTCCCAGAG	NM_001314054.1
***il-10***	CTGGACAACATACTGCTAACCG	AATGCTCCTTGATTTCTGGGC	NM_010548.2
***tnfα***	ACCCCTTTACTCTGACCCCTT	ACTGTCCCAGCATCTTGTGT	NM_001278601.1

##### Housekeeping gene stability analysis

**Fig. 1:**
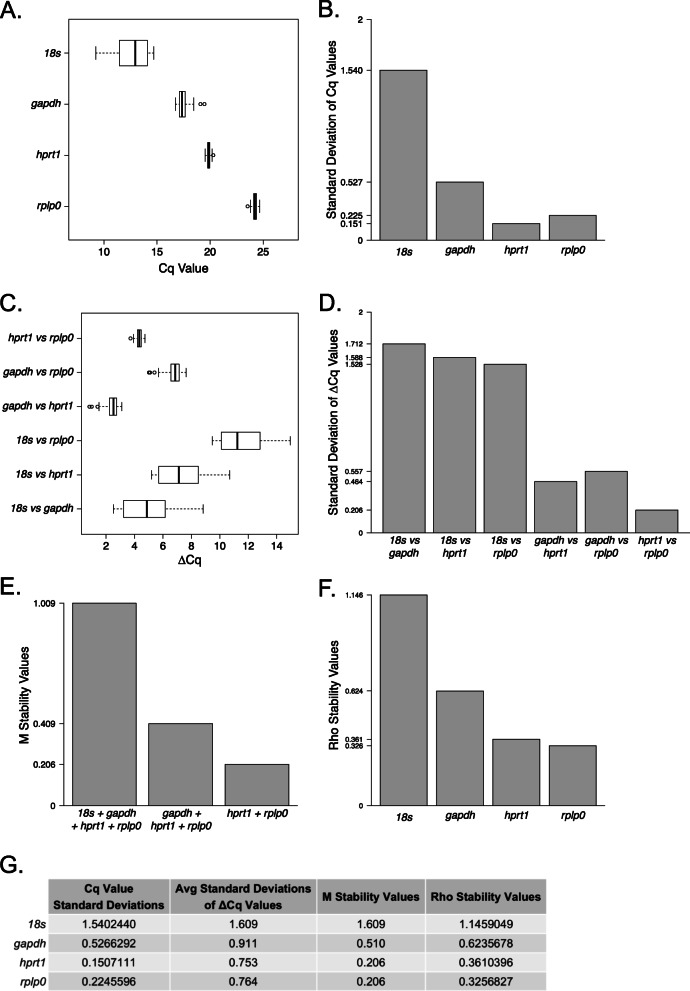
Housekeeping gene stability analysis. **A** Distribution of the quantification cycles (Cq) for housekeeping genes *18s*, *gapdh*, *hprt1*, and *rplp0*, with **B** associated standard deviations. **C** Distribution of the difference of Cq values (ΔCq) between pairs of housekeeping genes, and **D** the associated standard deviations. **E** Stability values calculated using gene ratio method by Vandesompele et al. 2002, which uses stepwise elimination of lowest stability (highest *M* value) to rank gene stability. **F** Stability values calculated using a model-based approach by Andersen et al. 2004 which measures expression variation such that highest stability results in the lowest Rho value. **G** Summary of results from each of the four methods of housekeeping gene stability are shown

To control for the transcriptional activity of the samples being analyzed, we confirmed the stability of four housekeeping genes (*18s*, *gapdh*, *hprt1*, and *rplp0*). While many studies use common housekeeping genes such as GAPDH or HPRT1, it is less common for authors to report that their chosen housekeeping gene is indeed stable across experimental groups or tissues prior to use in analyses. Thus, we confirmed the stability of our housekeeping genes using a combination of four techniques to ensure the most reliable quantification of gene expression in our studies. First, we assessed the variability of the candidate genes by measuring the standard deviation of the raw quantification cycle (Cq) values from all samples (Fig. 1A). We found that *18s* had the largest standard deviation of Cq values (1.540), followed by *gapdh* (0.527), *rplp0* (0.225), and *hprt1* (0.151; Fig. 1B). By this approach, *rplp0* and *hprt1* showed the greatest stability compared to *18s* and *gapdh*, with *hprt1* exhibiting the lowest variability.

Second, we employed a comparative ΔCq approach in which the standard deviations of the differences in Cq values (ΔCqs) between all possible pairs of candidate genes were compared [60] (Fig. 1C). From highest to lowest variability, the genes ranked as follows: *18s* (1.609 average standard deviation), *gapdh* (0.911), *rplp0* (0.764), and *hprt1* (0.753). Again, this method indicated that the most variable genes were *18s* and *gapdh* while the most stable genes were *rplp0* and *hprt1*, and this is most apparent when considering the lowest ΔCq standard deviation from this method was from the *rplp0* and *hprt1* comparison at 0.206 (Fig. 1D).

The third method we employed was that developed by Vandesompele and colleagues, which calculated the average pairwise variation of one candidate gene with all other candidate genes [61]. We used R packages ReadqPCR and NormqPCR [62] to calculate *M* stability values, as depicted in Fig. 1E. Consistent with the previous methods, *hprt1* and *rplp0* were the most stable of the candidate genes, with the lowest pairwise variability, *M* value, of 0.206.

Fourth, and last, we used a model-based stability analysis approach developed by Andersen et al., an algorithm called NormFinder (v5) [63]. This method protects against identifying two genes *via* the pairwise approach that might be misinterpreted as being the most stable if they are coregulated. Using this method, again, *hprt1* and *rplp0* were found to be the most stable genes with the lowest expression stability values (Fig. 1F). However, NormFinder resulted in *rplp0* having the lowest stability value of 0.326, indicating that the model-based approach identified *rplp0* as the most stable gene.

Together, these methods identified the two most stable candidate housekeeping genes as *hprt1* and *rplp0*. Vandesompele et al. [61] posits that using the geometric mean of multiple housekeeping genes results in more accurate expression levels of genes of interest. We calculated the geometric mean of the Cq values from *hprt1* and *rplp0* to be used in the 2^−ΔΔCq^ method for calculations of relative expression for our target genes.

##### Statistical analysis of mRNA gene expression

For each PCR reaction, the quantification cycle (Cq) was determined, and the 2^−ΔΔCq^ method was used to calculate the relative gene expression of each gene. Any samples with abnormal amplification curves, melt curves, and/or melt peaks across replicates were removed from analyses (*n* = 1/sex). Any outliers were identified as samples outside the range of 2 standard deviations from the group mean and excluded from analyses.

Baseline sex differences in relative gene expression (qPCR) were assessed by evaluating the male and female saline-treated groups. To directly and meaningfully compare these two groups in the PCR analysis, the male saline-treated group was normalized to the female saline-treated group and analyzed using independent, two-sample *t* tests.

To appropriately analyze sex differences in relative gene expression (qPCR) across the 24-h time course, we normalized each group to its respective same-sex saline-treated group to control for any sex differences in gene expression at baseline and used two-way ANOVA tests using treatment and sex as factors. Significant main effects and interactions (*p* < 0.05) were followed up using post hoc tests with Bonferroni corrections for multiple comparisons, and effect sizes were calculated using the partial eta squared method.

#### Multiplex assays

The second hemispheres of dorsal hippocampal tissue were processed as previously described using low-detergent RIPA buffer sonication [34]. Milliplex magnetic bead panel assays (CCL2, CXCL10, IFNγ, IL-1α, IL-1β, IL-2, IL-4, IL-6, and IL-10; Millipore Sigma, Burlington, MA) were used as per manufacturer’s instructions. Cytokine concentrations were calculated as pg/mg of hippocampal tissue *via* Luminex software. Only samples that showed readable bead counts according to the Luminex software were included in the analyses.

##### Statistical analysis of protein levels

Baseline sex differences in protein levels from multiplex assays were analyzed with independent, two-sample *t* tests comparing the saline-treated groups. To analyze changes in protein levels from poly I:C across the 24-h time frame, we used two-way ANOVA tests using treatment and sex as factors. Significant main effects and interactions (*p* < 0.05) were followed up using post hoc tests with Bonferroni corrections for multiple comparisons, and effect sizes were calculated using the partial eta squared method. Any outliers were identified as samples outside the range of 2 standard deviations from the group mean and excluded from analyses.

### Data visualization and statistical software

Data visualization and statistical analyses were completed using R 3.6.2 (R Core Team, 2019) with the following packages: dplyr (v0.8.5 [64];), tidyr (v1.0.2 [65];), rstatix (v0.5.0 [66];), DescTools (v0.99.34 [67];), sjstats (v0.17.9 [68];), ReadqPCR and NormqPCR [62], ggplot2 [69], gridExtra (v2.3 [70];), pheatmap (v1.0.12 [71];), and viridis (v0.5.1 [72];).

## Results

### Central administration of poly I:C induces physiological sickness responses

**Fig. 2:**
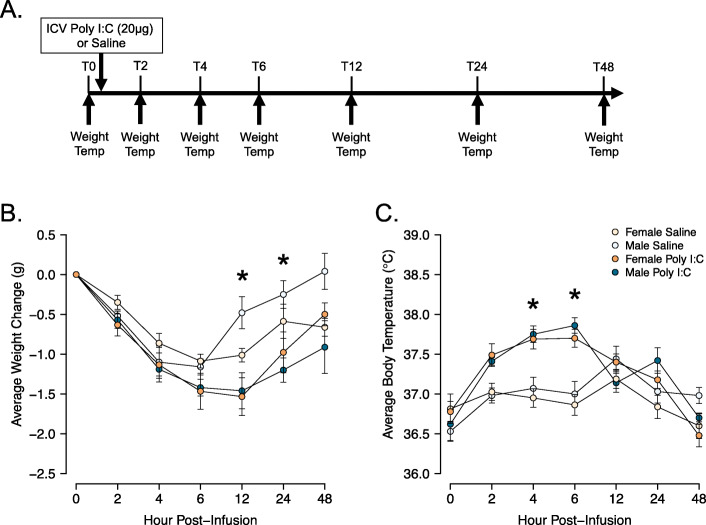
Analysis of sickness behaviors following poly I:C administration. **A** Timeline of body weight and temperature measurements following poly I:C or sterile saline administration. **B** Average weight change from baseline (Time = 0 h) prior to treatment. **C** Average body temperature as measured *via* rectal thermometer. Analyzed using mixed repeated-measures ANOVA. *p < 0.05 poly I:C- vs saline-treated groups

Both females and males showed physiological responses to poly I:C. Whereas both saline- and poly I:C-treated animals showed changes in weight across the 48-h period (Fig. 2B, main effect of Time: *F*(3.13, 96.92) = 28.899, *p* < 0.001, η^2^_*p*_ = 0.482), poly I:C caused weight loss in both sexes (main effect of Treatment: *F*(1, 31) = 8.781, *p* = 0.006, η^2^_*p*_ = 0.221; trend towards a Time × Treatment interaction: *F*(3.13, 96.92) = 2.476, *p* = 0.064, η^2^_*p*_ = 0.074). Specifically, males and females treated with poly I:C lost significantly more weight than the saline-treated animals at the 12- (*p* = 0.004) and 24-h (*p* = 0.022) time points. By 48 h post-treatment, the weights of poly I:C-treated animals had recovered and were no longer different from those of saline-treated animals (*p* = 1.00; Fig. 2B).

In both males and females, poly I:C caused significant increases in body temperature relative to the saline-treated group (Fig. 2C; main effect of Treatment: *F*(1, 31) = 23.759, *p* < 0.001, η^2^_*p*_ = 0.434; Time × Treatment interaction: *F*(4.6, 142.62) = 11.635, *p* < 0.001, η^2^_*p*_ = 0.273). Post hoc tests revealed that body temperature began to increase 2 h following poly I:C (*p* = 0.068), remained elevated at the 4- (*p* < 0.001) and 6-h (*p* < 0.001) time points, and recovered to saline-treated body temperatures by 12 h post-treatment (all *p* = 1.00; Fig. 2C). These data, and the similarity of febrile response in males and females, are consistent with previous studies using ICV [57] or systemic [73] poly I:C.

### Gene expression of hippocampal cytokines in response to poly I:C is greater in males compared with females

#### Glial activation markers

**Fig. 3:**
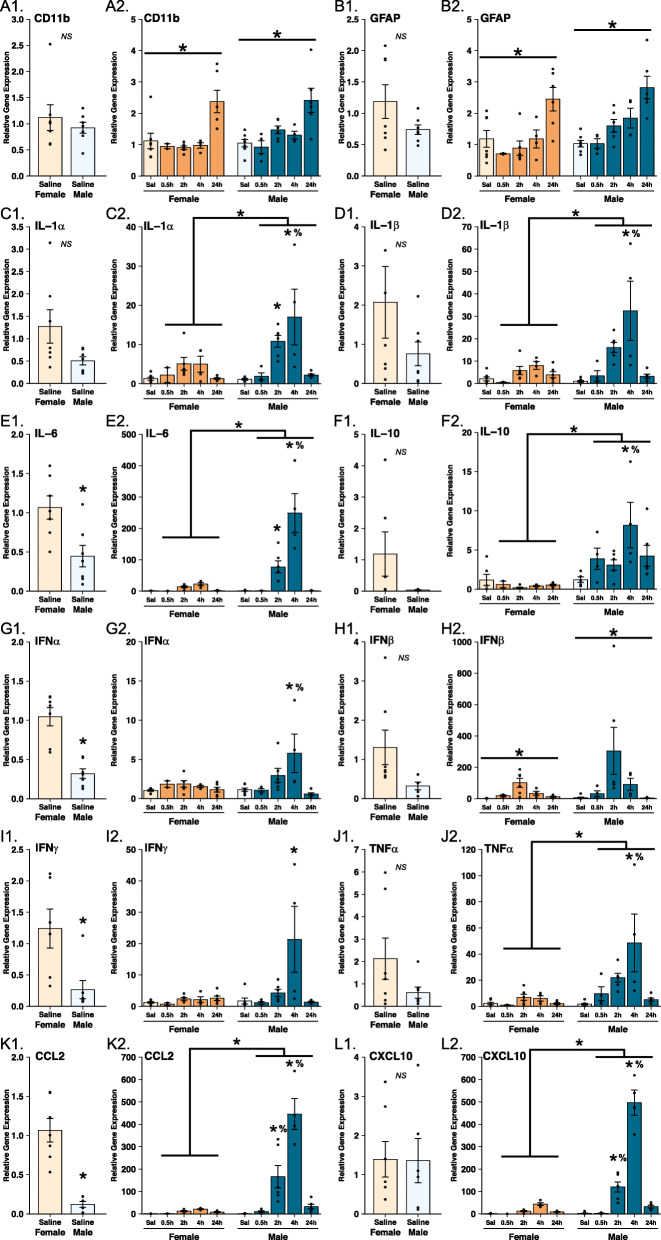
mRNA gene expression of cytokines, chemokines, and markers of glial activation in the hippocampus. Baseline gene expression was measured by normalizing the male saline-treated group to the female saline-treated group and analyzed using independent, two-sample *t* tests. Baseline expression of **A1** CD11b, **B1** GFAP, **C1** IL-1α, **D1** IL-1β, **E1** IL-6, **F1** IL-10, **G1** IFNα, **H1** IFNβ, **I1** IFNγ, **J1** TNFα, **K1** CCL2, and **L1** CXCL10 are shown. Gene expression changes following poly I:C treatment were calculated by normalizing time points after poly I:C treatment to the saline-treated groups within sex to eliminate confounding variables of baseline sex differences. Gene expression was analyzed using two-way ANOVA tests for **A2** CD11b, **B2** GFAP, **C2** IL-1α, **D2** IL-1β, **E2** IL-6, **F2** IL-10, **G2** IFNα, **H2** IFNβ, **I2** IFNγ, **J2** TNFα, **K2** CCL2, and **L2** CXCL10. * above a bracket covering both sexes indicates a significant main effect of sex (*p* < 0.05); * above a horizontal line covering just one sex indicates a significant main effect of treatment (*p* < 0.0.5); * above a single bar indicates a significant post hoc test (*p* < 0.05) vs the saline-treated group within sex; **%** above a single bar indicates a significant post hoc test (*p* < 0.05) vs females at the same time point

Poly I:C treatment significantly increased expression of both *cd11b* and *gfap*, although this appeared to be true only at the 24-h time point (Fig. 3A2, B2, respectively; *cd11b* main effect of Treatment: *F*(4, 42) = 12.96, *p* < 0.001, η^2^_*p*_ = 0.552; *gfap* main effect of Treatment: *F*(4, 42) = 12.992, *p* < 0.001, η^2^_*p*_ = 0.553). Sex did not affect the response of either *cd11b* or *gfap* to poly I:C (Sex × Treatment interactions: *cd11b*: *F*(4, 42) = 0.684, *p* = 0.607; *gfap*: *F*(4, 42) = 0.923, *p* = 0.460).

#### Interleukins

Poly I:C caused increased expression of *il-1α*, *il-1β*, and *il-6* in both males and females (Fig. 3C2, D2, E2, respectively; main effects of Treatment: *il-1α*: *F*(4, 42) = 9.784, *p* < 0.001, η^2^_*p*_ = 0.482; *il-1β*: *F*(4, 42) = 9.512, *p* < 0.001, η^2^_*p*_ = 0.475; *il-6*: *F*(4, 42) = 22.28, *p* < 0.001, η^2^_*p*_ = 0.680). In males, expression began to increase at the 2-h time point following poly I:C treatment for *il-1α* (*p* = 0.015; Fig. 3C2), *il-1β* (*p* = 0.057; Fig. 3D2), and *il-6* (*p* = 0.029; Fig. 3E2), showed peaks at the 4-h time point (*p* < 0.001 for all), and decreased to saline-treated levels by 24 h (*p* = 1.00 for all). Each of these genes also showed an overall greater expression in males than females (main effects of Sex: *il-1α*: *F*(1, 42) = 6.398, *p* = 0.015, η^2^_*p*_ = 0.132; *il-1β*: *F*(1, 42) = 6.695, *p* = 0.013, η^2^_*p*_ = 0.137; *il-6*: *F*(1, 42) = 21.1, *p* < 0.001, η^2^_*p*_ = 0.334), and a significantly greater magnitude of response in males compared with females (Sex × Treatment interactions: *il-1α*: *F*(4, 42) = 3.103, *p* = 0.025, η^2^_*p*_ = 0.228; *il-1β*: *F*(4, 42) = 4.288, *p* = 0.005, η^2^_*p*_ = 0.290; *il-6*: *F*(4, 42) = 15, *p* < 0.001, η^2^_*p*_ = 0.588). Post hoc tests revealed for all three genes, males exhibited an even greater response at only the 4-h time point compared with females (*p* < 0.05 for all). Notably, the peak *il-1α* and *il-1β* expression in males was roughly 3-fold higher than that of the peak female expression for these cytokines, and the *il-6* peak expression in males was more than 10-fold higher than that of females (Fig. 3C2, D2, E2, respectively).

Males showed greater *il-10* gene expression across all time points compared with females (Fig. 3F2; main effect of Sex: *F*(1, 39) = 25.642, *p* < 0.001, η^2^_*p*_ = 0.397). Additionally, poly I:C significantly increased gene expression of *il-10* in males, but not females (Sex × Treatment interaction: *F*(4, 39) = 3.304, *p* = 0.02, η^2^_*p*_ = 0.253). Specifically, male expression of *il-10* at the 4-h time point following poly I:C was significantly greater than that of saline-treated controls (*p* = 0.001), and this was also greater than the 4-h expression in females (*p* = 0.001; Fig. 3F2).

#### Interferons

Both *ifnα* and *ifnγ* showed a similar response pattern to poly I:C, whereby males treated with poly I:C exhibited a significant acute increase in gene expression of both cytokines, but females did not show the same response (Fig. 3G2, I2, respectively; *ifnα*: main effect of Treatment: *F*(4, 42) = 5.007, *p* = 0.002, η^2^_*p*_ = 0.323; Sex × Treatment interaction: *F*(4, 42) = 3.35, *p* = 0.018, η^2^_*p*_ = 0.242; *ifnγ*: main effect of Treatment: *F*(4, 40) = 4.698, *p* = 0.003, η^2^_*p*_ = 0.32; Sex × Treatment interaction: *F*(4, 40) = 4.178, *p* = 0.006, η^2^_*p*_ = 0.295). Specifically, 4 h after poly I:C treatment, males showed significantly elevated expression compared to the saline-treated controls (*ifnα: p* = 0.001; *ifnγ: p* = 0.0001), and this was greater in magnitude than the 4-h time point in females (*ifnα: p* = 0.014; *ifnγ: p* = 0.001; Fig. 3G2, I2, respectively). Female *ifnα* and *ifnγ* did not respond to poly I:C treatment at any time point.

In contrast, *ifnβ* showed a transient increase in both males and females, and there were no sex differences in magnitude of expression increase (Fig. 3H2; main effect of Treatment: *F*(4, 42) = 4.855, *p* = 0.003, η^2^_*p*_ = 0.316; Sex × Treatment interaction: *F*(4, 42) = 1.297, *p* = 0.287). Unlike all other cytokines examined in this study, peak expression appeared to be at the 2-h time point, and expression began decreasing again by 4 h post-treatment. The magnitude increase was also notable, with a 100-fold increase in females and a 300-fold increase in males.

#### Tumor necrosis factor alpha

Gene expression of *tnfα* increased in response to poly I:C, males had significantly higher expression than females overall, and males showed a greater magnitude of response compared with females (Fig. 3J2; main effect of Treatment: *F*(4, 42) = 6.407, *p* = 0.0004, η^2^_*p*_ = 0.379; main effect of Sex: *F*(1, 42) = 10.1, *p* = 0.003, η^2^_*p*_ = 0.194; Sex × Treatment interaction: *F*(4, 42) = 4.117, *p* = 0.007, η^2^_*p*_ = 0.282). Post hoc tests showed that males 4 h post-treatment had significantly greater expression than those treated with saline (*p* < 0.001), and this was again greater than the 4-h peak expression in females (*p* = 0.001; Fig. 3J2).

#### Chemokines

Poly I:C significantly increased the expression of both *ccl2* and *cxcl10* in males and females, with a peak increase in expression at 4-h post-infusion (Fig. 3K2, L2, respectively; main effects of Treatment: *ccl2*: *F*(4, 41) = 25.47, *p* < 0.001, η^2^_*p*_ = 0.713; *cxcl10*: *F*(4, 42) = 87.37, *p* < 0.001, η^2^_*p*_ = 0.893).

Expression of both *ccl2* and *cxcl10* was greater overall in males compared with females (Fig. 3K2, 3L2, respectively; main effects of Sex: *ccl2*: *F*(1, 41) = 44.55, *p* < 0.001, η^2^_*p*_ = 0.521; *cxcl10*: *F*(1, 42) = 92.79, *p* < 0.001, η^2^_*p*_ = 0.688); and males showed a markedly greater magnitude of response than did females for both chemokines (Sex × Treatment interactions: *ccl2*: *F*(4, 41) = 20.96, *p* < 0.001, η^2^_*p*_ = 0.672; *cxcl10*: *F*(4, 42) = 60.51, *p* < 0.001, η^2^_*p*_ = 0.852).

Remarkably, male *ccl2* expression peaked at nearly 450-fold greater than the expression of saline-treated males compared to a roughly 20-fold increased peak in females (Fig. 3K2). Similarly, *cxcl10* expression in males peaked at nearly 500-times that of saline-treated males while female *cxcl10* expression peaked at just over 40-times greater than saline-treated females (Fig. 3L2). These massive increases in gene expression are reflected in the strong effect sizes noted for the interaction effect above. Post hoc tests confirmed that the male 2- and 4-h time points post-treatment showed significantly greater gene expression of both *ccl2* and *cxcl10* than saline-treated males (Fig. 3K2, L2, respectively; *p* < 0.001). Additionally, both the male 2- and 4-h time points of both genes proved to be significantly greater than the 2- and 4-h time points in females, respectively (Fig. 3K2, L2, respectively; *p* < 0.01).

### Cytokine protein levels in males and females after poly I:C

#### Interleukins

**Fig. 4:**
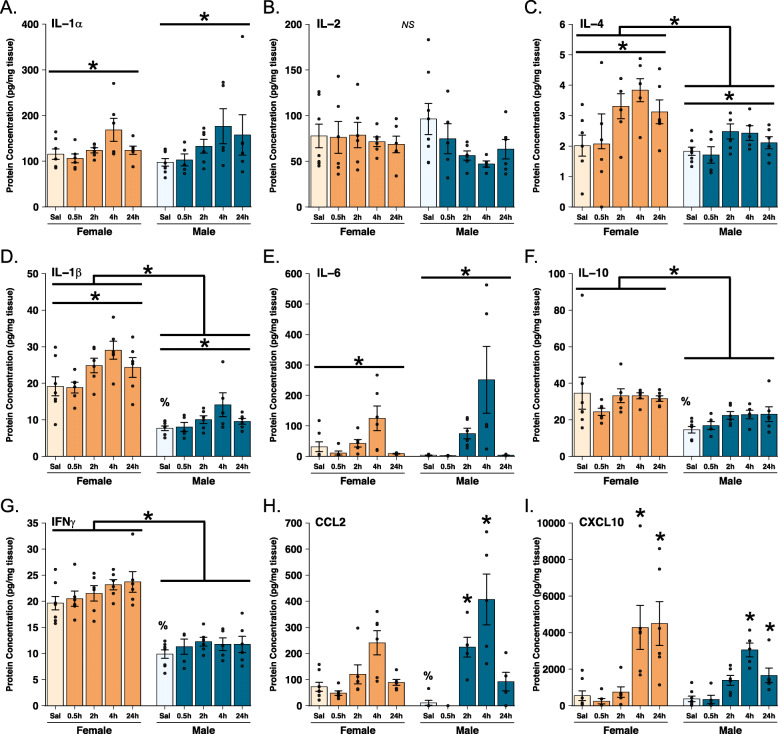
Protein levels of cytokines and chemokines in the hippocampus. Protein concentration (pg/mg) of **A** IL-1α, **B** IL-2, **C** IL-4, **D** IL-1β, **E** IL-6, **F** IL-10, **G** IFNγ, **H** CCL2, and **I** CXCL10 are shown. Two-way ANOVA tests were used to analyze these data. * above a bracket covering both sexes indicates a significant main effect of sex (*p* < 0.05); * above a horizontal line covering just one sex indicates a significant main effect of treatment (*p* < 0.5); * above a single bar indicates a significant post hoc test (*p* < 0.05) vs the saline-treated group within sex; **%** above a single bar indicates a significant post hoc test (*p* < 0.05) vs females at the same time point

IL-1α, IL-1β, IL-4, and IL-6 significantly increased following ICV poly I:C administration in both males and females (Fig. 4A–E, respectively; main effects of Treatment: IL-1α: *F*(4, 51) = 3.523, *p* = 0.013, η^2^_*p*_ = 0.216; IL-1β: *F*(4, 51) = 5.721, *p* = 0.001, η^2^_*p*_ = 0.31; IL-4: *F*(4, 51) = 5.146, *p* = 0.001, η^2^_*p*_ = 0.288; IL-6: *F*(4, 51) = 10.298, *p* < 0.001, η^2^_*p*_ = 0.447). In all cases, protein levels increase to a peak 4 h following poly I:C, similar to the effects seen in mRNA expression.

Both IL-4 and IL-1β also exhibited a main effect of sex such that protein levels of these cytokines, regardless of time point, were significantly higher in females compared with males (Fig. 4C, D, respectively; IL-4: *F*(1, 51) = 11.03, *p* = 0.002, η^2^_*p*_ = 0.178; IL-1β: *F*(1, 51) = 114.226, *p* < 0.001, η^2^_*p*_ = 0.691).

No interactions of sex and treatment were found for any of the interleukin cytokines examined here (Fig. 4A–E; IL-1α: *F*(4, 51) = 0.446, *p* = 0.775; IL-2: *F*(4, 51) = 0.987, *p* = 0.423; IL-4: *F*(4, 51) = 0.982, *p* = 0.426; IL-1β: *F*(4, 51) = 0.513, *p* = 0.726; IL-6: *F*(4, 51) = 1.779, *p* = 0.148).

Neither IL-2 nor IL-10 showed any effects of poly I:C treatment in either sex (Fig. 4B, F, respectively; main effects of Treatment: IL-2: *F*(4, 51) = 1.498, *p* = 0.217; IL-10: *F*(4, 51) = 1.122, *p* = 0.357). However, females had overall higher levels of IL-10 than did males (Fig. 4F; main effect of Sex; *F*(1, 51) = 20.27, *p* < 0.001, η^2^_*p*_ = 0.284).

#### Interferons

Unlike mRNA expression, IFNγ protein levels did not change following poly I:C administration in either sex (Fig. 4G; main effect of Treatment: *F*(4, 52) = 1.93, *p* = 0.119). However, IFNγ protein levels were higher in females relative to males (Fig. 4G; main effect of Sex: *F*(1, 52) = 150.64, *p* < 0.001; η^2^_*p*_ = 0.743). This was consistent with mRNA expression data where saline-treated females also showed significantly higher expression of *ifnγ* at baseline than did males (see Fig. 3I2).

#### Chemokines

Both CCL2 and CXCL10 were significantly increased in the hippocampus by ICV poly I:C and in different ways in males and females (Fig. 4H, I, respectively; CCL2: main effect of Treatment: *F*(4, 46) = 18.517, *p* < 0.001, η^2^_*p*_ = 0.617; Sex × Treatment interaction: *F*(4, 46) = 3.381, *p* = 0.017, η^2^_*p*_ = 0.227; CXCL10: main effect of Treatment *F*(4, 52) = 14.54, *p* < 0.001, η^2^_*p*_ = 0.528; Sex × Treatment interaction: *F*(4, 52) = 2.796, *p* = 0.035, η^2^_*p*_ = 0.177).

In males, CCL2 levels increased earlier (at 2 h) post-infusion than females (male saline vs 2 h *p* = 0.014; female saline vs 2 h *p* = 1.00; Fig. 4H). For CXCL10, females took longer for protein levels to begin to decrease as compared to the time course in males, with females still showing the massive elevation at 24 h post-infusion as they did at 4 h (Fig. 4I).

Notably, CCL2 and CXCL10 levels showed the most substantial increases out of all cytokines measured in protein analysis in the hippocampus. CCL2 levels induced by poly I:C peaked at approximately 4 times that of the saline-treated animals in females and nearly 8 times that of saline-treated males (Fig. 4H). For CXCL10 levels rose roughly 16-fold in females, and 12-fold in males after poly I:C administration (Fig. 4I).

### Baseline sex differences in mRNA expression and protein levels of select hippocampal immune molecules

Understanding baseline differences in neuroimmune gene expression and protein levels is essential for understanding sex differences in neuroimmune activation. We found that several cytokines and other immune markers showed greater than 2-fold higher levels at baseline (in saline-treated mice) in females compared with males, and in both gene expression and protein. In contrast, none of the markers examined here were higher in males than in females in either mRNA or protein levels at baseline. This is notable given that we observed the opposite pattern in activation, where males showed stronger poly I:C-induced activation of many cytokines.

#### Markers with significantly higher baseline levels in females compared with males

mRNA expression of *il-1α* exhibited a trend towards greater baseline expression in females (Fig. 3C1; *t*(12) = 2.006, *p* = 0.068), and *il-6* showed a significantly higher level in females compared with males (Fig. 3E1; *t*(11) = 3.079, *p* = 0.01, 95% CI [0.182, 1.062]). However, these gene expression differences were not reflected at the level of protein (Fig. 4A, E).

In contrast, although *il-1b* and *il-10* showed no difference in gene expression between the sexes (Fig. 3D1, F1, respectively; *il-1β*: *t*(12) = 1.365, *p* = 0.197; *il-10*: *t*(9) = 1.480, *p* = 0.173), females had significantly higher protein levels of both IL-1β and IL-10 than males (Fig. 4D, F, respectively; IL-1β: *t*(13) = 4.275, *p* = 0.001, 95% CI [5.682, 17.291]; IL-10: *t*(13) = 2.236, *p* = 0.044, 95% CI [0.672, 39.314]).

Two interferons (IFN), *ifnα* and *ifnγ*, also showed higher relative mRNA expression levels in females compared with males (Fig. 3G1 and I1: IFNα: *t*(12) = 5.546, *p* = 0.0001, 95% CI [0.441, 1.01]; IFNγ: *t*(11) = 2.995, *p* = 0.012, 95% CI [0.259, 1.694]). Likewise, protein levels of IFNγ were higher in saline-treated groups compared with males (Fig. 4G; *t*(14) = 6.475, *p* < 0.001, 95% CI [6.534, 13.006]).

Expression of chemokine *ccl2* also showed higher levels of both baseline gene expression (Fig. 3K1; *t*(12) = 3.287, *p* = 0.006, 95% CI [0.259, 1.279]), and protein levels (Fig. 4H; *t*(12) = 2.751, *p* = 0.018, 95% CI [12.798, 110.318]) in females compared with males.

#### Neuroimmune markers with no sex differences in baseline levels

Neither the microglial activation marker *cd11b* nor the astrocyte activation marker *gfap* showed sex differences in gene expression in the saline-treated groups (Fig. 3A1, B1, respectively; *cd11b*: *t*(12) = 0.723, *p* = 0.483; *gfap*: *t*(12) = 1.603, *p* = 0.135).

Levels of IL-2 and IL-4 protein did not differ between males and females (Fig. 3B, C, respectively, IL-2: *t*(12) = − 0.832, *p* = 0.420; IL-4: *t*(13) = 0.489, *p* = 0.633); nor were there differences in *tumor necrosis factor (tnf)α* gene expression (*t*(12) = 1.585, *p* = 0.139). Finally, CXCL10 did not differ between the sexes in either mRNA (Fig. 3L1; *t*(12) = − 0.923, *p* = 0.374) or protein (Fig. 4I; *t*(14) = 0.548, *p* = 0.592).

### Summary of mRNA and protein data

Overall, hippocampal mRNA expression and protein levels of most of the cytokines and chemokines examined in this experiment responded to central administration of poly I:C in both males and females. We found significant sex differences in baseline mRNA expression and protein levels of several cytokines, where females showed greater basal levels than males. In addition, we found the magnitude of mRNA expression increases was greater in males than females. Protein data showed this to be true only for 2 chemokines, CCL2 and CXCL10.

**Fig. 5:**
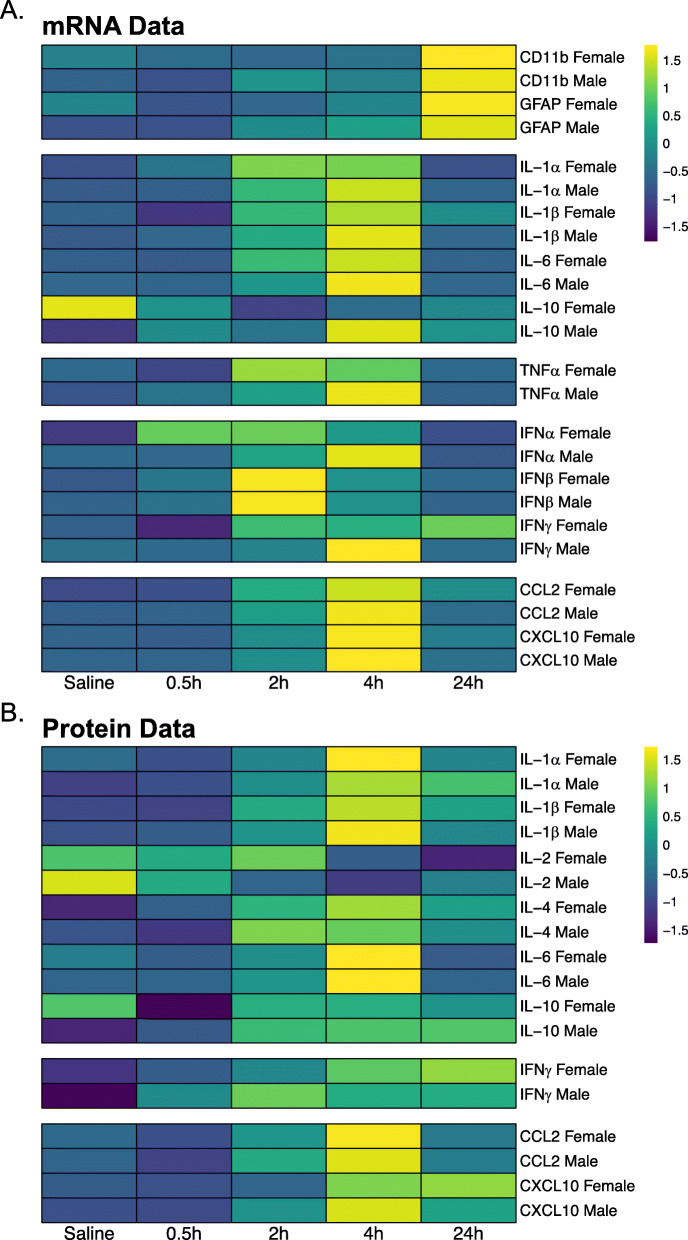
Heatmaps of gene expression and protein levels in the hippocampus. Changes in **A** mRNA gene expression and **B** protein levels for cytokines, chemokines, and markers of glial activation are shown. Values are centered and scaled across rows to highlight changes across the time course for each gene of interest; thus, differences in magnitude between genes are not depicted

The heatmaps shown in Fig. 5 indicate that most of the immune signaling molecules affected in the immediate phase following poly I:C treatment peaked at 4h for both mRNA expression (Fig. 5A) and protein levels (Fig. 5B) and returned to levels of saline-treated animals by 24 h post-infusion.

## Discussion

Here, we demonstrated that after central administration of poly I:C sufficient to induce acute physiological sickness (fever, weight loss) responses in both sexes, male and female mice showed acute hippocampal cytokine and chemokine elevations, as measured by both mRNA expression and protein levels, that followed the time course of fever. Interestingly, mRNA gene expression of *il-1α*, *il-1β*, *il-6*, *il-10*, *ifnα*, *tnfα*, *ccl2*, and *cxcl10* and protein levels of CCL2 and CXCL10 showed a stronger response in males compared with females. Further, gene expression of *il-10*, *ifnα*, and *ifnγ* increased in males only.

Poly I:C treatment in both sexes resulted in a significant and transient increase in hippocampal gene expression and protein levels of most, but not all, cytokines and chemokines measured, including IFNβ, IL-1α, IL-1β, IL-6, TNFα, CCL2, and CXCL10. That administration of an immune stimulant, including viral mimics such as poly I:C, induces a neuroimmune response is not new; however, most of the previous studies on poly I:C used peripheral administration [74–77]. As such, multiple, indirect mechanisms are likely involved in causing inflammation in the brain [41]. Peripheral administration of poly I:C, specifically, was found to induce neuroinflammation through a separate and distinct pathway than central administration [57]. Thus, ICV poly I:C administration is one way to study sex differences and similarities in the neuroimmune response to a TLR3 agonist, without initial interference from sex-specific peripheral response. Additionally, we extend previous work to include a broader set of cytokines and chemokines, including CCL2 and CXCL10, and type I interferons that typically respond to viruses. Given evidence of mechanistic complexities governing neuroimmune activation, particularly from stimulants such as poly I:C, and given that there are over 300 cytokines with important roles in the immune system and neural function, it is critical to begin looking beyond IL-1β, IL-6, and TNFα and more strongly consider implications of such limits in experimental design for the field of psychoneuroimmunology.

Males and females differ in immune responses, and the direction of these differences depends on whether one is looking in the periphery [13] or the brain [32, 33] and whether the immune challenge itself is systemic or brain-specific. We found that mRNA gene expression of *il-1α*, *il-1β*, *il-6*, *il-10*, *ifnα*, *tnfα*, *ccl2*, and *cxcl10* and protein levels of CCL2 and CXCL10 in the hippocampus showed a stronger response in males compared with females. A greater magnitude of cytokine and chemokine response in males is consistent with previous findings that male-derived astrocytes have a greater reaction to inflammatory insults compared with females [32, 33, 78, 79].

Poly I:C is recognized by microglia, astrocytes, and neurons *via* toll-like receptor 3 (TLR3) [80–82]. The interaction of these 3 cell types is crucial in mediating inflammatory responses [83, 84]. Given that TLR3 shows much greater expression in astrocytes relative to microglia [85], we speculate that the reaction of astrocytes in males may be driving the sex differences in magnitude gene expression response of cytokines following poly I:C. The astrocyte activation marker, GFAP, and the microglial activation marker, CD11b, did not increase until 24 h after poly I:C treatment and did not show sex differences. However, this does not absolve astrocytes or microglia from the acute response to poly I:C. Specifically, Norden and colleagues found that cytokine gene expression from both astrocytes and microglia preceded increases in astrocyte and microglial activation markers, (GFAP and Iba1, respectively), and that these activation markers similarly did not show reliable increases until the 24-h time point [86]. Further work is needed to understand how neuroimmune cells, and in particular astrocytes, drive sex differences in cytokine response to poly I:C.

We observed that for most cytokines examined here, males showed a greater response to poly I:C than did females. Whereas others have reported increases in select inflammatory markers following poly I:C treatment, these studies used either only used male [75, 76] or female rodents [74, 77]. To the best of our knowledge, this is the first direct comparison of hippocampal cytokines in males and females as a consequence of poly I:C. Whether the greater magnitude in male response to poly I:C indicates greater neuroprotection or vulnerability to cognitive dysfunction is yet to be determined.

A critical question, arising from our observation of greater baseline mRNA expression and protein levels of cytokines and chemokines in females relative to males, is what is the biological relevance of these differences, and how do they relate to activated neuroimmune states? One possibility is that females mount a greater immune response to help clear viral loads and recover faster [38, 87–89], and also start out with greater immune activity that allows them to reach necessary activation states faster than males. Perhaps females do not need to have as strong of an activated response because they already have “more players in the game”. This layer of nuance for understanding sex differences in immune/neuroimmune function adds to the broader notion that sex differences are not just about who has a stronger response, but that the type and pattern of response matters [26, 34], together with the context (e.g., dose, type of challenge, timing, hormonal states [13, 34, 88, 90]) all of which contribute to the complexity of understanding sex differences and their functional implications. Future work will need to address whether and how sex differences in the cytokine and chemokine basal levels or activation in response to immune challenge result in modulation of neural function and contribute to sex-biases in neurological and psychiatric disease.

Of particular note, we observed a sex-specific pattern of expression of the interferon family of cytokines in the hippocampus. Specifically, males showed increases in IFNα, IFNβ, and IFNγ, but females only showed a significant response in IFNβ. This is consistent with previous findings that showed increased gene expression of IFNβ, but not IFNα, in females in response to peripheral poly I:C, though this study did not measure these effects in males for comparison [77]. Type I interferons, IFNα and IFNβ, are key to the anti-viral response of the immune system and, as such, are known to respond to viral stimulants including poly I:C [54, 91–94]. Consistent with our data, in which IFNβ showed an early peak expression levels, type I interferon activity is responsible for inducing inflammatory cytokines such as IL-6 and TNFα [75, 77]. Additionally, interferon signaling from poly I:C treatment also results in altered glutamatergic signaling [91, 92], which is critical for hippocampal memory formation [95]. One caveat is that we only measured IFNα and IFNβ gene expression. Nevertheless, other studies have demonstrated a correspondence of increased IFNβ gene expression and modulation of memory in females [58, 77]. Thus, given that males show increased expression of both IFNα and IFNβ in the hippocampus following poly I:C whereas females only induce IFNβ, together with the roles of IFNβ in learning and memory, interferon-related signaling is likely key for understanding sex differences in virus, or virus-like, modulation of memory and cognition.

This study characterized the neuroimmune and sickness responses to central administration of poly I:C, and we observed sex-specific patterns of hippocampal cytokine transcription and translational responses. Specifically, we identified type I interferons as one potential node mediating sex-specific cytokine responses and neuroimmune effects on synaptic plasticity and cognition. Additionally, the magnitude of response of cytokines such as CCL2 and CXCL10 highlight the importance of future work incorporating a more comprehensive set of inflammatory markers using multiple endpoints. Neuroimmune activation is known to play a role in cognitive deficits and affective dysregulation in diseases such as Alzheimer’s disease and other dementias [96], Post-traumatic stress disorder [97, 98], depression [11, 99], and now also COVID-19 [100]. Given the sex/gender biases in prevalence, severity, and/or survival outcomes, identifying sex-specific neuroimmune responses will provide novel targets for personalized prevention and treatment of these diseases.

## Data Availability

The data used and analyzed for the current study are available from the corresponding author upon reasonable request.

## Abbreviations

CCL2: C-C motif chemokine ligand 2
Cd11b: Cluster of differentiation molecule 11b
CXCL10: C-X-C motif chemokine ligand
COVID-19: Coronavirus disease 2019
GFAP: Glial fibrillary acidic protein
ICV: Intracerebroventricular
IFN: Interferon
IL: Interleukin
LPS: Lipopolysaccharide
mRNA: Messenger ribonucleic acid
Poly I:C: Polyinosinic:polycytidylic acid
qPCR: Quantitative polymerase chain reaction
TNF⍺: Tumor necrosis factor ⍺
